# Factors Related to Visual Outcomes after Lens Surgery in Isolated Microspherophakia

**DOI:** 10.1155/2022/9089203

**Published:** 2022-06-15

**Authors:** Jialei Zheng, Lina Cheng, Zexu Chen, Tianhui Chen, Yongxiang Jiang

**Affiliations:** ^1^Eye Institute and Department of Ophthalmology, Eye & ENT Hospital, Fudan University, Shanghai 200031, China; ^2^NHC Key Laboratory of Myopia (Fudan University), Key Laboratory of Myopia, Chinese Academy of Medical Sciences, Shanghai 200031, China; ^3^Shanghai Key Laboratory of Visual Impairment and Restoration, Shanghai 200031, China; ^4^Shaanxi Provincial Clinical Research Center for Ophthalmology Diseases, First Hospital of Xi'an, Xi'an, Shaanxi 710000, China; ^5^Shaanxi Key Laboratory of Ophthalmology, Shaanxi Institute of Ophthalmology, Xi'an, Shaanxi, China

## Abstract

**Purpose:**

To evaluate the main factors influencing visual performance after lens subluxation surgery in subjects with isolated MSP.

**Design:**

Retrospective study.

**Methods:**

In this study, 38 eyes of subjects with isolated MSP (microspherophakia) were included and divided into two groups based on preoperative IOP (intraocular pressure), IOP <21 mmHg, or IOP ≧21 mmHg. Phacoemulsification and scleral-fixated modified capsular tension ring implantation were performed with or without goniosynechialysis according to the IOP. Some ocular biometric parameters, such as corneal curvature, corneal pachymetry, endothelial cell count (ECC), anterior chamber depth (ACD), and axial length, were evaluated. The best-corrected visual acuity (BCVA) and IOP of these subjects were measured before the surgery and during <1 month and 3- to 6-month postoperative follow-ups.

**Results:**

Compared with the high IOP group, the normal IOP group was significantly younger and had better preoperative BCVA, a higher ECC, deeper ACD, a lower postoperative IOP, and flatter total corneal refractive power K1. The multivariable analysis revealed that preoperative ACD (*b* = −0.113, *t* = −2.070, *P*=0.047) and preoperative BCVA (*b* = 0.153, *t* = 2.562, *P*=0.015) were significantly associated with postoperative BCVA at 3–6 months. A preoperative ACD of 1.86 mm was found to be the optimal cut-off point for 3- to 6-month postoperative BCVA of ≧20/63 (≤0.52 logMAR).

**Conclusions:**

In addition to the effect of normal IOP, better preoperative BCVA and deeper ACD also correlated with better visual outcomes after lens surgery. Preoperative ACD served as a warning for isolated MSP subjects, especially for the risk of irreversible loss of postoperative vision. This trial is registered with “ChiCTR2000039132”.

## 1. Introduction

Microspherophakia (MSP) is a relatively rare condition in which the lens is smaller and more spherical than usual. The zonular fibers of MSP become weak and lack tension, which directly leads to the emergence of small spherical crystals [1]. According to previous reports [2], approximately 33% of subjects with MSP have incomplete dislocation and 9% have complete dislocation of the lens, and 20% of these subjects are blinded due to secondary glaucoma. During follow-up, the blinding rate increases to 30% [2]. Due to the gradual progressive relaxation of the zonular fibers and the severity of damage to vision in MSP, the surgical effect could be various, which depends on the disease stages. Therefore, it is ideal if lens surgery is performed before the occurrence of secondary glaucoma to avoid irreversible damage caused by high IOP (intraocular pressure) on the optic nerve.

According to existing case reports, the following biological parameters may have specific effects on MSP. Because the spherical lens leads to a decreased anterior chamber depth (ACD), the ACD of MSP ranges from 0.55 to 2.87 mm [3, 4]. The mean anteroposterior distance of the lenses ranges from 4.06 to 6.75 mm [4–7]. The axial length (AL) varies from 21.5 mm to 25 mm, and keratometry varies between 39.4 D and 45.6 D [5–8]. Muralidhar et al. [9] reported that half of MSP subjects had high IOP, and glaucoma developed in 44.4% of the subjects' eyes. Therefore, these may be used as specific biological parameters for evaluating the pathological stage of MSP. However, there is no scientific evidence describing the effectiveness of the biological parameters for assessing isolated MSP.

In our study, we compared how the biometric characteristics of age, corneal curvature, endothelial cell count (ECC), ACD, AL, preoperative and postoperative best-corrected visual acuity (BCVA), and IOP affect the outcome of surgery in isolated MSP. The influence of these factors and postoperative BCVA on these associations was further explored. Moreover, the principal factors driving vision loss distribution were also discussed. This study provides evidence of the high-risk stage of isolated MSP in the clinic.

## 2. Methods

### 2.1. Subject Selection

This is a retrospective study. Isolated MSP was diagnosed based on the methods of Chan RT [10]. The inclusion criteria were as follows. (1) Diagnostic criteria: bilateral involvement; lenticular myopia; after mydriasis, the equatorial edge of the lens could be seen under slit lamp or operating microscope; whole lens zonular fibers were found to be sparse and lax by ultrasound biomicroscopy; and the dislocation of lens can be seen in supine position. (2) The surgeries were completed successfully. (3) Those who completed the 1-month and 3–6-month follow-ups. Exclusion criteria were as follows. (1) Lenses fell into the anterior chamber or vitreous cavity. (2) History of ocular trauma. (3) History of other ophthalmic surgery, such as congenital cataract, retinal detachment, epiretinal membrane, or antiglaucoma surgery. (4) Those with systemic associations such as Marfan syndrome, Alport syndrome, homocysteinuria, Weill–Marchesani syndrome, etc. (5) Follow-ups were not completed, or IOP and BCVA data were missing.

Isolated MSP treated between July 2018 and February 2021 in the Eye and ENT Hospital of Fudan University were involved in the study; we enrolled 24 subjects (38 eyes). The subjects were divided into high IOP group and normal IOP group according to whether the initially diagnosed IOP was greater than 21 mmHg. Written informed consent was obtained from all participants or guardians of children. The study was approved by the Human Research Ethics Committee of the Eye and ENT Hospital of Fudan University and adhered to the tenets of the Declaration of Helsinki (ChiCTR2000039132).

### 2.2. Measurement of Ocular Biometric Parameters

The biometric parameters were collected before surgery. We measured the anterior corneal curvature (mean keratometry, Km), ACD, and AL by partial coherence interferometry (IOLMaster 500 & 700, Carl Zeiss Meditec AG, Jena, Germany). The corneal pachymetry, anterior and posterior corneal curvature, and total corneal refractive power (TCRP) were assessed by rotating Scheimpflug camera (Pentacam, Oculus Optikgeräte GmbH, Wetzlar, Germany). During the postoperative visits at <1 and 3–6 months, BCVA was measured by the same experienced ophthalmologist with a comprehensive refractometer (NIDEK ARK 510, Japan). The noncontact tonometer (CT-80, Topcon Medical Systems, Japan) was used to measure the IOP. The logarithm of the minimum angle of resolution (logMAR) was used to describe BCVA. The data for all subjects were the average of measurements taken three times.

### 2.3. Surgical Technique

**Figure 1:**
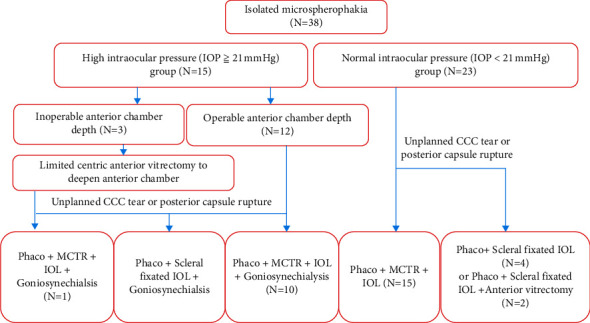
Subjects' surgery flow chart. IOP: intraocular pressure; phaco: phacoemulsification; CCC: continuous curvilinear capsulorhexis; MCTR: modified capsular tension rings; IOL: intraocular lens.

All surgeries of phacoemulsification (Phaco), goniosynechialysis, scleral-fixated modified capsular tension ring (MCTR) implantation, and sutured scleral fixation of an intraocular lens (IOL) were performed by an experienced doctor (Dr. YX Jiang) [11]. A 2.6 mm superior corneal incision was made after general anesthesia. A continuous curvilinear capsulorhexis (CCC) of 4.0–5.0 mm was applied manually. The capsular bag was suspended and fixed with four capsular hooks (CapsuleCare, Med Devices Lifesciences, India). In adult subjects, the stop and chop technique was performed to deal with nuclei over grade three. In children, the lens material was removed with a phacoemulsifer (Alcon Laboratories Inc, USA) using irrigation/aspiration under a low vacuum with a reduced bottle height of 65 cm. The MCTR (Morcher GmbH, Germany) was then sutured to the sclera 1.5–2 mm behind the limbus with 9-0 polypropylene (MANI Inc. Japan) using a modified knot-free z-suture technique. After removal of the capsular hooks, an IOL was delivered into the capsular bag. In the high IOP group, if the ACD <1.50 mm, it was too shallow to operate, and limited centric anterior vitrectomy was performed via the pars plana to decrease vitreous pressure, facilitating the injection of viscoelastic agent into the anterior chamber to deepen the anterior chamber at the beginning of the surgery. The peripheral part of the iris was pulled towards the center and back 360° with capsulorhexis forceps, and the adhesions of the angle were separated twice. Then, the viscoelastic agent was used to separate the anterior chamber angle again at 360°. If there was vitreous leakage into the anterior chamber, anterior vitrectomy was required. In the case of incomplete CCC and rupture of the posterior capsule, an IOL was fixed to the sclera with 9-0 polypropylene using the modified knotless *z*-suture technique [12]. The surgery flow chart for the subjects is shown in Figure 1. In the first week after the surgery, the subjects were treated with sodium hyaluronate, praprofen, and ofloxacin eye drops and prednisone acetate ophthalmic suspension 1% four times a day. The frequency was changed to three times a day in the 2nd week and reduced to two times a day in the 3rd week, and we continued to use this frequency for 1 month.

### 2.4. Statistical Analysis

Statistical analyses were performed with SPSS version 23.0 software (IBM Corp. Armonk, NY, USA). A Kolmogorov–Smirnov test of normality was performed for all variables. Absolute frequency (*n*) and relative frequency (%) were used to describe categorical variables. The statistical results for the continuous variables included the mean ± standard deviation (SD) and median P_50_ (P_25_, P_75_) according to the normality of the data. The Student' s *t*-test and Wilcoxon rank-sum test (Mann–Whitney *U*-test) were used, as appropriate, to compare continuous data. The Chi-square test and Fisher's test were used to compare categorical variables. Univariate linear regression analysis was used to evaluate correlations between ocular biometric parameters and postoperative BCVA. Multivariable linear regression analysis was performed to identify the predictors of postoperative BCVA at 3–6 months. The receiver operating characteristic (ROC) curve was used to evaluate the high-risk values of the preoperative ACD and preoperative BCVA. A *P* value of <0.05 was considered significant.

## 3. Results

### 3.1. Preoperative Characteristics of All Subjects

This study included 38 eyes from 24 isolated MSP subjects treated between July 2018 and February 2021 at the Eye & ENT Hospital of Fudan University. The mean subjects age was 27 ± 19.24 years. The basic preoperative parameters of these eyes are shown in Table 1.

### 3.2. Surgical Outcomes

**Figure 2:**
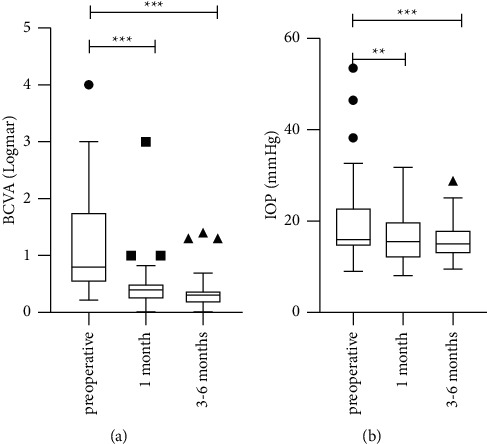
BCVA: best-corrected visual acuity; IOP: intraocular pressure. (a) Changes of BCVA in all subjects. (b) Changes of IOP in all subjects. ^*∗*^*P* < 0.05; ^*∗∗*^*P* < 0.01; ^*∗∗∗*^*P* < 0.001.

Scleral suture fixation IOL surgery was performed in 10 eyes and MCTR implantation in 28 eyes. The mean BCVAs (logMAR) of the 38 eyes with MSP were 1.22 ± 1.0 (preoperative), 0.46 ± 0.49 (1 month), and 0.39 ± 0.33 (3–6 months). The mean IOPs (mmHg) were 20.2 ± 9.8 (preoperative), 15.62 ± 5.46 (1 month), and 16.92 ± 4.65 (3–6 months). The preoperative vs. postoperative differences in BCVA and IOP were statistically significant (*P* < 0.05). There was an improvement in BCVA and a reduction in IOP at 1-month and 3–6-month follow-ups compared with the preoperative measurements, but no significant differences between the 1-month follow-up and 3–6-month follow-up were observed (*P*=0.102). The results are shown in Figure 2.

### 3.3. Postoperative Complications

Posterior capsular opacification (PCO) was found in five eyes at follow-up. When the IOP was 21–30 mmHg (five eyes) after the surgery, one antiglaucoma eye drop was added to reduce the IOP. When the IOP was 30–40 mmHg (three eyes), two drugs were used. After the IOP reached a normal value, the drugs were gradually reduced. In the process of drug reduction, changes in the IOP were monitored. During the 3–6-month follow-up, six eyes still needed antiglaucoma drugs to control IOP. There was no endophthalmitis, retinal detachment, or dislocation of the IOL by the end of follow-up.

### 3.4. Differences between High IOP and Normal IOP Groups

The eyes were divided into two groups according to IOP. The normal IOP group was allocated based on a preoperative IOP of <21 mmHg without any IOP-lowering drugs and glaucoma surgery. The other subjects were entered into the high IOP group. There were 23 eyes in the normal IOP group and 15 eyes in the high IOP group. Differences between the two groups are summarized in Table 2. There were significant differences in age, preoperative BCVA, preoperative IOP, ECC, preoperative ACD, and preoperative TCRP K1. During the follow-up at 3–6 months, there were significant differences in postoperative IOP between the two groups.

### 3.5. Univariate and Multivariable Analyses of Various Factors Associated with Postoperative BCVA at 3–6 Months

Univariate analysis of various factors (Table 3) associated with postoperative BCVA at 3–6 months revealed that preoperative BCVA, preoperative and postoperative IOP at 3–6 months, and preoperative ACD were significantly associated with postoperative BCVA. In the multivariable analysis (Table 4), preoperative BCVA and preoperative ACD were the variables significantly associated with the 3–6-month postoperative BCVA.

### 3.6. The Relationship between Preoperative ACD and 3–6-Month Postoperative BCVA ≧20/63 (0.52 LogMAR)

**Figure 3:**
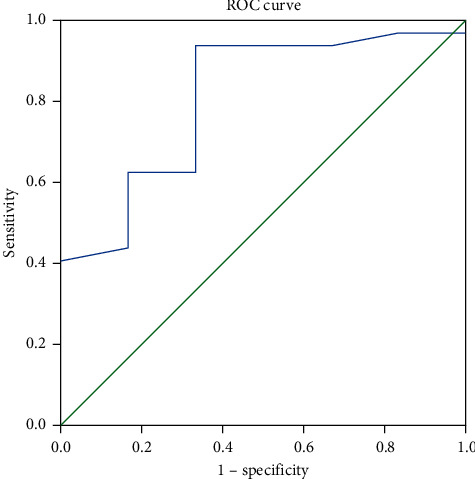
ROC curve for preoperative ACD in relation to the postoperative BCVA at 3–6 months. ROC: receiver operating characteristic; ACD: anterior chamber depth; BCVA: best-corrected visual acuity.

The ROC curve was analyzed to consider the potential risk values for preoperative ACD that resulted in a 3–6-month postoperative BCVA of ≧20/63 (≤0.52 LogMAR), as shown in Figure 3. The area under the curve was 0.807. The value of preoperative ACD at 1.86 mm was found to be the optimal cut-off point for a 3–6-month postoperative BCVA of ≧20/63 (≤0.52 logMAR). The preoperative ACD presented a sensitivity of 93.8% and a specificity of 66.7% (*P*=0.018).

## 4. Discussion

Subjects with MSP usually have poor vision and complications such as high refractive myopia, lens subluxation, glaucoma, corneal decompensation, and retinal detachment [9, 10]. The severity of the MSP determines the differences in postoperative visual function and prognosis of these subjects [9]. Therefore, this paper mainly discusses the application of MCTR in MSP and the factors affecting visual performance after surgery.

Due to the particularity of lens morphology and the complexity of the complications, the treatment methods used for MSP are variable [4, 13–15]. The abnormal lens should be removed surgically [13]. Lensectomy, anterior vitrectomy, and glaucoma surgery are common choices for managing secondary glaucoma in MSP [16]. However, the former surgical method tends to have more postoperative complications [17]. In Jarrete's study [17], including 166 dislocation lens cases, lens extraction was done in 114 eyes in the series. Vitreous was lost in 47 (41%) at surgery, and 12 retina detachments (11%) occurred following extraction of the dislocated lens. With the rapid development of phacoemulsification and lens capsule stabilizing devices, posterior chamber (PC) IOLs are also implanted with capsular tension rings (CTR) or capsular tension segments (CTS) [18]. The postoperative visual acuity recovered better, and the incidence of postoperative complications decreased. Yang et al. [4] did a 3-year research including 19 subjects with microspherophakia and glaucoma, 7 eyes underwent phacoemulsification and CTR, whereas 17 eyes underwent lensectomy with scleral-fixated posterior chamber (PC) IOL implantation. The postoperative BCVA increased from 0.79 ± 0.36 to 0.44 ± 0.38 (logMAR) in the CTR group and from 1.15 ± 0.75 to 0.43 ± 0.38 (logMAR) in the lensectomy group at the 3-year follow-up. Six eyes (85.7%) in the CTR group developed different degrees of posterior capsular opacity. However, the follow-up of other complications was not reported.

MCTR has also been recognized as providing a fixed support for capsular bags with zonular dialysis [14]. Cionni et al. [19] reported a postoperative BCVA of 20/40 or better in 88.9% of subjects with congenital loss of zonular support by phacoemulsification with MCTR implantation, while the mean follow-up was 14.6 months (range 2 to 32 months). The incidence of retinal detachment (1%), mild persistent iritis (3.3%), and suture spontaneously broke (10.0%) was low.

With the support of the MCTR, capsular hooks [20, 21], and other new technology, our lens surgery of choice for isolated MSP has changed from lens extraction, which has more complications, to phacoemulsification and MCTR implantation, with fewer complications. In this study, we used MCTR implantation (28 eyes, 73.7%) and IOL scleral interlaminar surgery (10 eyes, 26.3%). Goniosynechialysis was carried out for subjects with high IOP. In our study group, 78.9% of subjects at 3- to 6-month follow-up achieved a vision of 0.48 logMAR or better, compared with 61.1% of subjects in previous studies in which lensectomy was done by the limbal route or by pars plana, while the mean follow-up was 8.55 ± 3.98 years [9]. The preoperative vs. postoperative differences in BCVA and IOP were statistically significant.

The findings suggest these surgical methods were effective. One month after the surgery, the visual performance of all eyes was significantly improved, and the improvement tended to be stable at 3- to 6-month follow-up. Compared with previous studies [17, 19], our surgery also had small probabilist intraocular complications. Thus, the surgical procedure appeared safe over the short-term observation period.

Which factors are the main influences on visual performance in isolated MSP still needs to be determined. We further explored certain parameters; the surgical method; the occurrence of PCO which would affect the postoperative BCVA. As a result, the surgical method and PCO might not be a key index for determining the postoperative visual outcome.

In our study, 39.5% (15/38) of subjects with isolated MSP had a high IOP, which is comparable to 44% of subjects with MSP seen in previous studies [9]. Due to abnormal relaxation of the lens zonular fibers in MSP [22], lens dislocation or subluxation may occur, and the small lens is often displaced towards the anterior chamber, which may lead to shallowing of the ACD and the loss of corneal endothelial cells [23]. When the iris contacts the anterior surface of the lens repeatedly over a long time period, it causes repeated pupil block, resulting in the closure of angle adhesion and finally the formation of chronic angle-closure glaucoma [24]. This process occurs repeatedly and leads to continuous glaucoma development. Compared with the high IOP group, the normal IOP subjects were younger and had better preoperative BCVA, a higher ECC, a greater anterior chamber depth, lower postoperative IOP, and flatter TCRP K1. Therefore, these biological parameters should be important and meaningful in subjects with high IOP. During the 3- to 6-month follow-up period, six eyes still needed to be treated with two to three hypotensive drugs to maintain the IOP below 21 mmHg. Therefore, the alteration of the structure and function of the trabecular meshwork caused by the spherical lens was partly irreversible, suggesting that surgery should be performed before excessive goniosynechia. However, there was no significant linear correlation between the high/normal IOP group and postoperative BCVA. As a result, finding more sensitive and specific indicators to predict postoperative BCVA would be beneficial.

In this study, univariable and multivariable linear regression of various factors revealed that the preoperative ACD and BCVA were significantly associated with postoperative BCVA at 3–6 months, which suggested subjects with deeper ACD and better preoperative BCVA could achieve a better postoperative visual outcome. The ACD and BCVA measurements are easy to obtain on ophthalmic examination. The disadvantage of BCVA is that it is considered to be a subjective index, and crystal astigmatism, corneal astigmatism, and refractive amblyopia might also affect the results of optometry. Therefore, we did not take preoperative BCVA to be an evaluation parameter. On the other hand, preoperative ACD is an objective indicator that is measured directly by partial coherence interferometry, and it has good practicability and operability in the clinic. The results of the study showed that ACD can reflect the severity of the spherical lens and predict postoperative visual outcomes. We used a visual outcome of less than 20/63 as the diagnostic value because the diagnostic criterion of low vision is 20/63 according to the World Health Organization (WHO) criteria [25]. A preoperative ACD of 1.86 mm was the optimal cut-off point for poor postoperative vision. This result reminded us that we should not only pay attention to the IOP of isolated MSP but also closely monitor the preoperative ACD, and surgical measures should be performed in time to avoid the occurrence of poor vision.

There were some limitations to this study. Firstly, only 24 subjects were enrolled; therefore, the sample size was rather small. Secondly, some of the follow-up periods were rather short and variable due to the COVID-19 pandemic in 2020. Therefore, the final follow-up results from 3 to 6 months after surgery were selected, instead of more exact time points, for the endpoint analysis. Thirdly, our findings might be biased because of the retrospective methodology and the lack of a control group for comparison.

In conclusion, phacoemulsification and scleral-fixated MCTR implantation for isolated MSP subjects was safe and effective in the short term. During the follow-up of isolated MSP, we also need to pay attention to changes in the BCVA and ACD in addition to the IOP. The critical value for the preoperative ACD could be used as a feasible reference to avoid the occurrence of low visual ability after the surgery. In future studies, it will be important to extend the follow-up time to observe the long-term complications of these surgical methods and the subsequent changes in BCVA and IOP.

## Figures and Tables

**Table 1:** Baseline characteristics of total subjects.

	Total
Subjects/eyes	24/38
Sex (female : male)	14 (58.33%) : 10 (41.67%)
Eyes (right : left)	18 (47.37%) : 20 (52.63%)
Age (years)	27 ± 19.24 (4∼60)
Preoperative BCVA (logMAR)	1.22 ± 1.00 (0.22∼4)
Preoperative IOP (mmHg)	20.21 ± 9.81 (9.0∼53.5)
High IOP/normal IOP	15 (39.47%) : 23 (60.53%)
Central ECC (cells/mm^2^)	2733.861 ± 475.9466 (1468∼3818)
Corneal pachymetry (**μ**m)	546.39 ± 46.39 (463∼622)
Preoperative AL (mm)	24.82 ± 2.22 (21.99∼30.14)
Preoperative SimK (D)	42.78 ± 1.99 (29.4∼47.4)
Preoperative TRCP K_m_ (D)	43.04 ± 1.86 (39.2∼46.2)
Preoperative ACD_ext_ (mm)	2.76 ± 0.87 (1.01∼4.62)
B/F ratio	78.72 ± 16.28 (77.4∼85.7)

BCVA: best-corrected visual acuity; IOP: intraocular pressure; ECC: endothelial cell count; WTW: white to white; AL: axial length; km: mean keratometry; TCRP: total corneal refractive power; ACD: anterior chamber depth; B/F ratio: mean radius of the posterior corneal surface/mean radius of the anterior corneal surface ratio; SD: standard deviation.

**Table 2:** Differences of high IOP group and normal IOP group.

Characteristics	Normal IOP group	High IOP group	*P* value
Sex (female/male)	13 (56.52%)/10 (43.48%)	6 (40.0%)/9 (60.0%)	0.254
Eyes (right/left)	12 (52.17%)/11 (47.83%)	5 (33.33%)/10 (66.67%)	0.254
Age (years)	21.65 ± 19.90 (4∼60)	35.2 ± 15.38 (15∼59)	**0.032** ^*∗*^
Preoperative BCVA (logMAR)	0.7 ± 0.51 (0.22∼2.70)	2.02 ± 1.06 (0.5∼4.0)	**<0.001** ^*∗*^
Preoperative IOP (mmHg)	15.33 ± 2.5 (11.0∼20.7)	27.36 ± 12.07 (9∼53.5)	**<0.001** ^*∗*^
Central ECC (cells/mm^2^)	2969.0 ± 413.06 (2248∼3818)	2404.67 ± 351.47 (1468∼2776)	**<0.001** ^*∗*^
Corneal pachymetry (*μ*m)	541.26 ± 46.50 (503∼612)	554.267 ± 38.3210 (526∼605)	0.374
Preoperative AL (mm)	24.84 ± 2.07 (22.0∼28.24)	24.84 ± 2.52 (21.99∼30.14)	0.995
Preoperative ACD (mm)	3.12 ± 0.77 (1.51∼4.80)	2.31 ± 0.81 (1.05∼3.65)	**0.006** ^*∗*^
Preoperative KI (D)	41.44 ± 6.74 (39.89∼48.49)	43.65 ± 1.71 (40.53∼46.40)	0.255
Preoperative K2 (D)	44.01 ± 2.29 (41.01∼48.84)	44.61 ± 1.7 (42.29∼46.96)	0.413
Preoperative SimK (D)	42.30 ± 1.94 (39.4∼47.4)	43.53 ± 1.88 (40.5∼46.4)	0.060
Preoperative TCRP K1 (D)	41.81 ± 1.69 (39.2∼45.5)	43.26 ± 1.92 (41.1∼46.2)	**0.019** ^*∗*^
Preoperative TCRP K2 (D)	43.33 ± 2.05 (40.4∼47.3)	44.28 ± 1.65 (41.8∼46.4)	0.141
Preoperative TCRP Km (D)	42.58 ± 1.81 (40.2∼46.1)	43.75 ± 1.75 (41.1∼46.0)	0.055
B/F ratio	82.59 ± 2.53 (77.4∼85.7)	82.27 ± 1.60 (78.8∼84.9)	0.682
Postoperative IOP (mmHg) <1 month	13.91 ± 3.81 (10.1∼19.0)	18.07 ± 6.43 (10.5∼31.0)	0.059
Postoperative BCVA (logMAR) <1 month	0.33 ± 0.18 (0.04∼0.69)	0.65 ± 0.75 (0∼3)	0.193
Postoperative IOP (mmHg) (3–6 months)	13.64 ± 2.97 (10.6∼22.5)	19.59 ± 4.13 (14.0∼29.1)	**<0.001** ^*∗*^
Postoperative BCVA (logMAR) (3–6 months)	0.26 ± 0.13 (0∼0.52)	0.53 ± 0.47 (0.04∼1.39)	0.124

BCVA: best-corrected visual acuity; IOP: intraocular pressure; ECC: endothelial cell count; WTW: white to white; AL: axial length; Km: mean keratometry; TCRP: total corneal refractive power; ACD: anterior chamber depth; B/F ratio: mean radius of the posterior corneal surface/mean radius of the anterior corneal surface ratio.

**Table 3:** Univariate analysis of factors associated with postoperative BCVA at 3–6 months.

Various factors	Beta coefficient (95% CI)	*t* value	*P* value
Age (years)	−0.005 (−0.01, 0.001)	−1.755	0.088
Preoperative BCVA (logMAR)	0.204 (0.115, 0.292)	4.650	**<0.001** ^*∗∗∗*^
Preoperative IOP (mmHg)	0.019 (0.009, 0.029)	3.883	**<0.001** ^*∗∗∗*^
Central ECC (cells/mm^2^)	0.000 (0.000, 0.000)	−1.474	**0.015** ^*∗*^
Corneal pachymetry (*μ*m)	0.001 (−0.001, 0.004)	1.085	0.285
Preoperative ACD (mm)	−0.229 (−0.346, −0.113)	−4.021	**<0.001** ^*∗∗∗*^
Preoperative AL (mm)	0.188 (−0.022, 0.078)	1.150	0.258
Preoperative km (D)	−0.035 (−0.091, 0.020)	−1.294	0.204
Preoperative TCRP Km (D)	−0.249 (−1.106, 0.014)	−1.542	0.132
B/F ratio	−0.004 (−0.053, 0.051)	−0.026	0.979
MCTR implantation/suture-fixated IOL	−0.190 (−0.434, 0.054)	−1.581	0.123
PCO	−0.081 (−0.409, 0.247)	−0.501	0.619
Postoperative IOP (mmHg) ≤1 month	0.254 (−0.010, 0.044)	1.315	0.200
Postoperative IOP (mmHg) 3–6 months	0.397 (0.006, 0.053)	2.561	**0.015** ^*∗*^

CI: confidence interval; BCVA: best-corrected visual acuity; IOP: intraocular pressure; ECC: endothelial cell count; ACD: anterior chamber depth; AL: axial length; km: mean keratometry; TCRP: total corneal refractive power; B/F ratio: mean radius of the posterior corneal surface/mean radius of the anterior corneal surface ratio; MCTR: modified capsular tension ring; IOL: intraocular lens; PCO: posterior capsular opacification. ^*∗*^: *P* < 0.05; ^*∗∗*^: *P* < 0.01; ^*∗∗∗*^: *P* < 0.001.

**Table 4:** Multivariate analysis of factors associated with postoperative BCVA at 3–6 months.

Various factors	Beta coefficient (95% CI) (*N* = 38)	*T* value	*P* value
Preoperative BCVA (logMAR)	0.153 (0.031, 0.275)	2.562	**0.015** ^*∗*^
High/normal IOP	−0.093 (−0.376, 0.191)	−0.665	0.511
Preoperative ACD (mm)	−0.113 (−0.225, 0.002)	−2.070	**0.047** ^*∗*^
Postoperative IOP (mmHg) 3–6 months	0.018 (0.008, 0.044)	1.436	0.161

TCI: confidence interval; BCVA: best-corrected visual acuity; IOP: intraocular pressure; high IOP: preoperative IOP ≧21 mmHg; normal IOP: preoperative IOP <21 mmHg; ACD: anterior chamber depth. ^*∗*^: *P* < 0.05; ^*∗∗*^: *P* < 0.01; ^*∗∗∗*^: *P* < 0.001.

## Data Availability

Data are available in a public, open-access repository. All data relevant to the study are included in the article or uploaded as supplementary information.
